# Toxic Effects of Waterborne Pb Exposure on Hematological Parameters and Plasma Components in Starry Flounder, *Platichthys stellatus*

**DOI:** 10.3390/ani15070932

**Published:** 2025-03-24

**Authors:** Min-Jung Kim, Kyung Mi Lee, Sung-Pyo Hur, Cheol Young Choi, Jun-Hwan Kim

**Affiliations:** 1 Department of Marine Life Science, Jeju National University, Jeju 63243, Republic of Korea; zwillinge06@naver.com (M.-J.K.); hursp@jejunu.ac.kr (S.-P.H.); 2 Aquaculture Industry Division, West Sea Fisheries Research Institute, National Institute of Fisheries Science, Incheon 22383, Republic of Korea; bioykm@korea.kr; 3 Division of Marine BioScience, National Korea Maritime and Ocean University, Busan 49112, Republic of Korea; 4 Department of Aquatic Life Medicine, Jeju National University, Jeju 63243, Republic of Korea

**Keywords:** Pb exposure, LC_50_, hematological parameters, plasma components, starry flounder

## Abstract

Lead (Pb) is a non-essential toxic metal that accumulates in aquatic environments, negatively impacting fish health. This study evaluated the acute toxicity of Pb in starry flounders (*Platichthys stellatus*). Fish (41 ± 8.1 g, 14 ± 0.9 cm) were exposed to Pb concentrations of 0, 10, 20, 40, 80, 160, 320, and 640 mg Pb^2+^/L for 96 h. The lethal concentration (96 h LC_50_) was determined to be 227 mg Pb^2+^/L. Hematological analysis showed significant decreases in RBC counts, hemoglobin, and hematocrit, while MCH and MCHC increased at ≥160 mg Pb^2+^/L. Plasma calcium levels significantly decreased following Pb exposure, and AST activity was reduced. These findings suggest that acute waterborne Pb exposure adversely affects survival, hematological parameters, and plasma components in *P. stellatus*, providing insight into Pb toxicity in aquatic organisms.

## Simple Summary

Lead pollution in water can harm fish health and survival. This study examined the effects of lead exposure on starry flounders (*Platichthys stellatus*) over 96 h. Fish exposed to high lead levels showed increased mortality, with half dying at 227 mg/L. Lead also reduced red blood cell counts and hemoglobin, affecting oxygen transport. Additionally, calcium levels dropped, which may impact bone health. However, glucose and protein levels remained stable. These results suggest that while starry flounders tolerate lead better than some fish, exposure still poses health risks. Understanding these effects can help protect aquatic life from pollution.

## 1. Introduction

Heavy metals (HMs) are toxic environmental pollutants that pose significant risks to ecosystems and living organisms. Their interactions in the aquatic environment can influence their bioavailability, toxicity, and potential for bioaccumulation in organisms [1]. Among these, lead (Pb), unlike essential trace metals such as copper (Cu), zinc (Zn), iron (Fe), and manganese (Mn) which are involved in biochemical reactions in aquatic organisms, has no biochemical function; it is a non-essential metal that does not have any metal and can be highly toxic even at low concentrations [2]. In general, Pb can exist naturally in rock walls, soil, and water, and it may be introduced into the aquatic ecosystem and exist in trace amounts due to natural phenomena such as erosion caused by volcanic activity and geological weathering [3]. Recently, Pb has been used in various industrial activities such as mining, smelting, batteries, and paint manufacturing due to its low melting point, high plasticity, and corrosion resistance [4,5]. Excessive usage of Pb in many industrial activities can lead to discharge into water at high levels, and high concentrations of waterborne Pb exposure can be fatally toxic to aquatic life [6].

High levels of Pb exposure in water are primarily ingested by fish through the food chain, rather than through the gills. This can lead to significant shifts in the fish’s intestinal bacterial flora [7]. Once in the circulatory system, Pb ions accumulate in major active organs, inhibiting proper growth by damaging the liver and deteriorating digestive function [8,9]. In addition, continuous Pb exposure to aquatic animal lead reduces the ion reabsorption activity in the fish body, resulting in the loss of calcium, an essential ion, which can cause osmotic dysregulation; it also affects the mucus cells distributed in the gill epithelial layer, leading to excessive mucus secretion, which may result in respiratory failure and suffocation [10]. Continuous waterborne Pb exposure can cause neurological disorders such as hyperventilation and hyperactivity and can also induce deformities such as scoliosis and affect reproductive activity and reproduction [11,12]. In individuals spawned by mothers continuously exposed to waterborne Pb, it can cause persistent learning and memory impairments, as well as behavioral disorders along with neurological damage for at least three generations [13].

Pb causes histological lesions in the liver, kidneys, and major tissues in fish, and high levels of waterborne Pb exposure can cause mass mortality of aquatic animals [14]. This study was conducted to determine the toxic effects on aquatic animals due to exposure to toxic substances in the aquatic ecosystem. Indicators of mortality following acute exposure are essential [15]. Lethal concentration 50% (LC_50_) is the concentration at which 50% of test animals experience mortality due to exposure to toxic substances and is a widely used indicator to evaluate the effects of various toxic substances present in water [16,17]. Fish are a major ecological indicator species for evaluating the effects of toxic exposure in the aquatic ecosystem [18]. In this study, the LC_50_ of fish due to waterborne Pb exposure was used as an important indicator to determine vulnerability to and tolerance limit of Pb toxicity in fish.

Pb that enters the fish body by exposure to Pb in the water can penetrate the blood through the gills and intestinal epithelial cells, which can directly affect the fish’s circulatory system and hematopoietic tissue, resulting in changes in structure and deterioration of function [19]. The presence of Pb in fish blood not only impairs the oxygen-carrying ability of the blood by reducing the volume or number of red blood cells but also inhibits the binding of red blood cells to heme synthase, thereby reducing the erythrocyte neogenesis and causing deformation and membrane destruction of erythrocyte [20]. Hematological properties are an indicator of the health status in fish and can be affected by physiological and seasonal factors, such as the age and nutritional status [21]. In particular, exposure to toxic substances can directly affect hematological properties by causing physiological or pathological changes in fish [22]. Therefore, changes in hematological parameters and plasma components due to exposure to waterborne Pb can suggest a major indicator for evaluating the toxic effects on fish by exposure to waterborne Pb.

Starry flounder, *Platichthys stellatus*, is a cold-water fish species widely distributed in all seas, including the North Pacific coast, and is one of Korea’s representative aquaculture fish [23,24]. The *P. stellatus* is a euryhaline fish species that can live in brackish water areas, as it has excellent osmoregulation ability and salt tolerance [25]. In addition, *P. stellatus* has strong resistance to diseases, so their survival rate against disease infection is relatively high compared to other fish species. Recently, they have been known as a high-quality fish species, and their economic value is continuously increasing as demand increases [26]. Meanwhile, waterborne Pb that presents at high levels in the aquatic ecosystem can act as fatal toxicity to the aquatic ecosystem and cause mass mortality [27]. However, little research has been conducted on the toxic effects and toxicity standard indicators of *P. stellatus* by waterborne Pb exposure. Therefore, the purpose of this study was to evaluate the physiological toxic effects through changes in hematological parameters and plasma components of the *P. stellatus* by waterborne Pb exposure and to present standard indicators for waterborne Pb toxicity. The findings provide critical data on the lethal concentration (96 h LC_50_) and key biomarkers that can serve as standard indicators for assessing Pb toxicity in aquatic environments.

## 2. Materials and Methods

### 2.1. Experimental Fish and Environment

The *P. stellatus* (mean weight 41 ± 8.1 g, mean total length 14 ± 0.9 cm) used in this experiment were purchased from a fish farm near Pyoseon in Jeju Island and reared in a laboratory environment. They were acclimatized in a laboratory environment for 2 weeks before the experiment. The *P. stellatus* were fed food twice a day during the acclimatization period in a 1000 L circular filtration-type aquarium, and the photoperiod was maintained at a light condition of 12 h light and 12 h dark (12L/12D). The experimental tank was a 30 L glass square tank, and acute exposure was conducted for 96 h in 8 concentration groups (0, 10, 20, 40, 80, 160, 320, and 640 mg Pb^2+^/L). The experiment was carried out with acute exposure using a total of 48 experimental fish (8 Pb concentration groups × 6 fish per experimental group) randomly selected from among healthy individuals. Water quality (water temperature, dissolved oxygen, salinity, and pH) were measured using a portable water quality analyzer (YSI-Professnal plus; YSI Inc., Yellow Springs, OH, USA), and ammonia, nitrite, nitrate, and phosphate were measured using the Marine Environmental Process Testing Method (Ministry of Oceans and Fisheries). The measured values are presented in Table 1. In this study, acute exposure to lead was conducted using a standard stock solution of 100,000 mg Pb^2+^/L, which was prepared using lead nitrate [Pb(NO_3_)_2_, Lead(II) nitrate]. The exposure was carried out according to the designated concentration in each tank.

### 2.2. Lethal Concentration 50% (LC_50_)

To determine sublethal concentrations due to waterborne lead exposure, mortality was checked for each tank at 0, 1, 3, 6, 12, 24, 48, 72, and 96 h after Pb exposure, and mortality individuals were removed immediately upon observation. After 96 h, the lethal concentration 50% value was calculated using a statistical program (SPSS Inc., Chicago, IL, USA, probit model) based on the final mortality of individuals by exposure to waterborne Pb.

### 2.3. Hematological Parameters

For hematological analysis, blood was collected from fish 96 h after exposure to waterborne Pb. Blood was sampled from 6 animals from the control group exposed to 120 mg Pb^2+^/L, and 5 fish were sampled at 240 mg Pb^2+^/L. Blood was collected using a syringe treated with heparin (Sigma Chemical, St. Louis, MO, USA), and hemoglobin, hematocrit, and red blood cell count (RBC count) were analyzed immediately after blood collection. Hemoglobin levels were measured by the cyan-methemoglobin method using a clinical kit (Asan Pharm. Co., Ltd., Seoul, Republic of Korea). Hematocrit was measured by pouring blood into a capillary, centrifuging at 12,000 rpm for 10 min in a micro-hematocrit centrifuge (hanil, Gimpo, Republic of Korea), and using a micro-hematocrit reader. RBC count was performed by diluting the blood 400 times with Hayem’s solution and then counting it under an optical microscope using a hemo-cytometer (Marienfeld Superior, Lauda-Königshofen, Germany).

Based on the results for hemoglobin, hematocrit, and RBC count, the mean corpuscular volume (MCV), mean corpuscular hemoglobin (MCH), and mean corpuscular hemoglobin concentration (MCHC) were calculated in the following manner.

MCV (µL) = Hematocrit (%)/RBC count (10^7^/µL) × 10

MCH (pg) = Hemoglobin (g/dL)/RBC count (10^7^/µL) × 10

MCHC (%) = Hemoglobin (g/dL)/Hematocrit (%) × 100

### 2.4. Plasma Components

To analyze changes in plasma components due to exposure to waterborne Pb, the collected blood was centrifuged at 3000× *g* for 15 min at 4 °C to separate the plasma samples. Calcium and magnesium were measured as plasma inorganic components. Calcium was measured using the OCPC method and magnesium was measured using a clinical kit (Asan Pharm. Co., Ltd., Seoul, Republic of Korea) according to the Xylidyl blue-I method. Glucose, cholesterol, and total protein were measured as plasma organic components. A commercially available clinical kit (Asan Pharm. Co., Ltd., Seoul, Republic of Korea) was used for glucose by the GOD/POD method, cholesterol by the colorimetric method, and total protein by the Biuret method. AST (aspartate aminotransferase) and ALP (alkaline phosphatase) were measured as plasma enzyme activities. AST was analyzed by the Reitman–Frankel method at 505 nm, and ALP was analyzed by the King–King method at 500 nm using a clinical kit (Asan Pharm. Co., Ltd., Seoul, Republic of Korea).

### 2.5. Statistical Analysis Method

In this experimental analysis, 6 fish were used in each concentration, and all experiments were conducted in 3 repeated analyses. The experimental analysis results were considered statistically significant when *p* < 0.05 through Tukey’s multiple range test by conducting an ANOVA test using the SPSS/PC+ statistical package software version 20 (SPSS Inc., Chicago, IL, USA).

## 3. Results

### 3.1. Survival Rate and Lethal Concentration (LC_50_)

**Figure 1:**
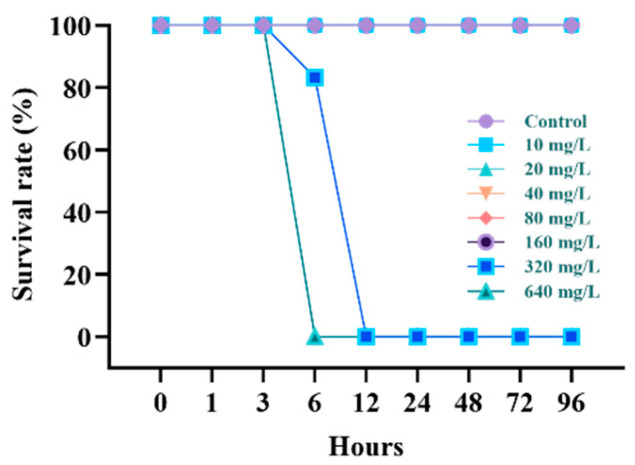
Survival rate of starry flounder, *Platichthys stellatus*, exposed to waterborne lead for 96 h.

The survival rate of the *P. stellatus* after acute exposure to waterborne Pb is shown in Figure 1. In the control group, no dead individuals were exposed to 160 mg Pb^2+^/L, and when exposed to 320 mg Pb^2+^/L, dead individuals began to occur from 6 h after exposure, and 100% died after 12 h. When exposed to 640 mg Pb^2+^/L, 100% died after 6 h. The lethal concentration 50 (LC_50_) of the *P. stellatus* after waterborne Pb exposure is demonstrated in Table 2. The LC_50_ of the *P. stellatus* after waterborne Pb exposure was 227 mg Pb^2+^/L.

### 3.2. Hematological Parameters

**Figure 2:**
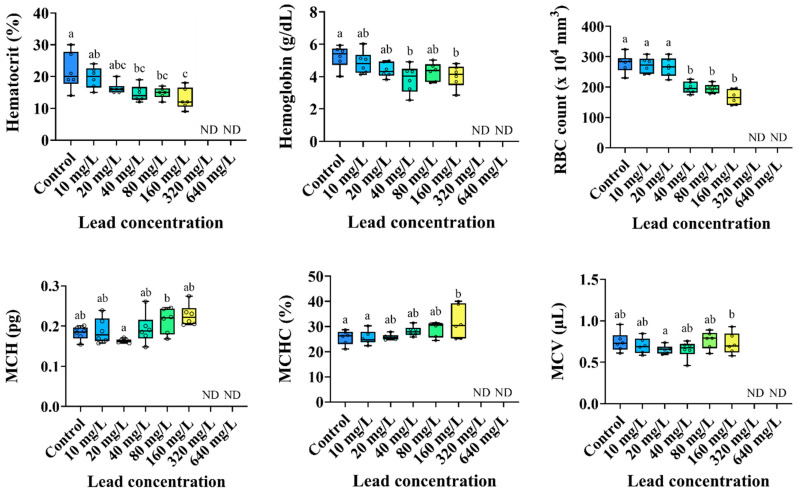
Hematological parameters of starry flounder, *Platichthys stellatus*, exposed to waterborne lead for 96 h. Values with different letters indicate significant difference at 96 h (*p* < 0.05) after one-way ANOVA following Tukey’s multiple range test.

The hematological parameters of the *P. stellatus* according to acute exposure to waterborne Pb are shown in Figure 2. The hemoglobin concentration, hematocrit value, and RBC count of the *P. stellatus* were significantly decreased by waterborne lead exposure of 40 mg Pb^2+^/L or more (*p* < 0.05). Based on the results for hemoglobin, hematocrit, and RBC count, MCV (µL) did not show significant changes. MCH (pg) also tended to increase by waterborne Pb exposure of 10 mg Pb^2+^/L or more, but no significant changes were observed. MCHC (%) significantly increased by waterborne Pb exposure of 160 mg Pb^2+^/L or more (*p* < 0.05).

### 3.3. Plasma Components

**Figure 3:**
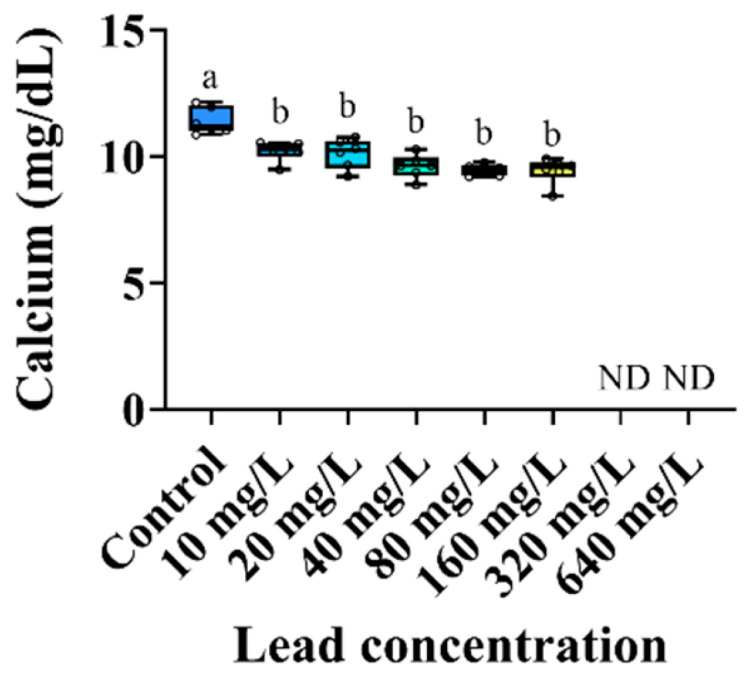
Inorganic plasma component of starry flounder, *Platichthys stellatus*, exposed to waterborne lead for 96 h. Values with different letters indicate significant difference at 96 h (*p* < 0.05) after one-way ANOVA following Tukey’s multiple range test.

**Figure 4:**
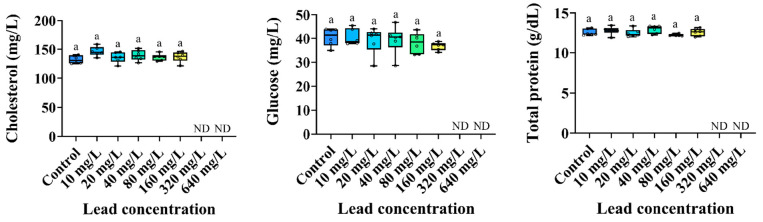
Organic plasma components of starry flounder, *Platichthys stellatus*, exposed to waterborne lead for 96 h. Values with different letters indicate significant difference at 96 h (*p* < 0.05) after one-way ANOVA following Tukey’s multiple range test.

**Figure 5:**
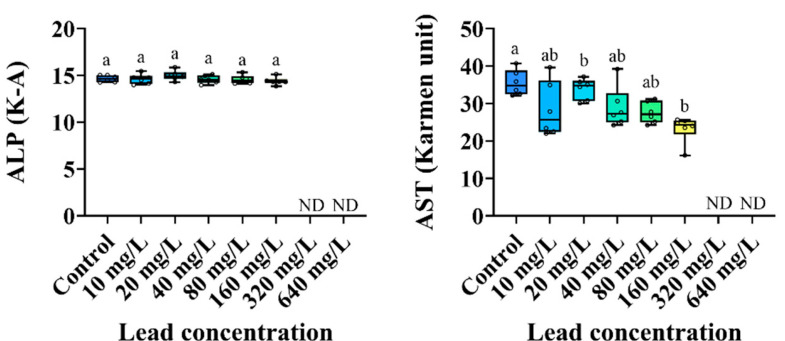
Enzymatic plasma components of starry flounder, *Platichthys stellatus*, exposed to waterborne lead for 96 h. Values with different letters indicate significant difference at 96 h (*p* < 0.05) after one-way ANOVA following Tukey’s multiple range test.

The changes in the plasma inorganic components of the *P. stellatus* by acute exposure to waterborne Pb are shown in Figure 3. The plasma calcium level of the *P. stellatus* after acute exposure to waterborne Pb showed a significant decrease (*p* < 0.05). The plasma organic components of the *P. stellatus* after acute exposure to waterborne Pb are shown in Figure 4. The plasma glucose showed a decreasing trend, but no significant change was observed. The plasma cholesterol and plasma total protein did not show significant changes by waterborne Pb exposure (*p* < 0.05). The plasma enzyme components of the *P. stellatus* by waterborne Pb exposure are shown in Figure 5. The plasma AST showed a significant decrease at concentrations of 20 mg Pb^2+^/L or more after waterborne Pb exposure, but the plasma ALP did not show a significant change (*p* < 0.05).

## 4. Discussion

Pb is generally present in trace amounts of 0.03~0.05 g/L in aquatic ecosystems, but it can be introduced into water in large quantities by excessive industrial and agricultural activities [28]. High levels of Pb in water can accumulate in the gills of fish, causing damage, deformation, and dysfunction in the gill epithelium, leading to death by hypoxia [29]. According to Nekoubin et al. [30], the 96 h LC_50_ of Caspian roach, *Rutilus rutilus caspicus*, following acute exposure to waterborne Pb was reported to be 276.167 mg Pb^2+^/L, which is similar to the results of this study, and Al-Kshab and Yehya (2021) reported that the 96 h LC_50_ of mosquitofish, *Gambusia affinis*, was 50.514 mg Pb^2+^/L [31]. Abedi et al. (2012) also reported that the 96 h LC_50_ for acute exposure to waterborne Pb in common carp, *Cyprinus carpio*, and sutchi catfish, *Pangasius hypophthalmus*, was 77.33 mg Pb^2+^/L and 48.06 mg Pb^2+^/L, respectively, showing significant differences among fish species, which was due to species-specific characteristics of each fish species, such as waterborne Pb tolerance, skin thickness, and the presence or absence of scales for absorption through the body surface [32]. Abdullah et al. (2017) reported significant age-related differences in waterborne Pb toxicity in Rohu, *Labeo rohita*, at 30, 60, and 90 days of age that were exposed to waterborne Pb above the tolerance limit, with LC_50_ of 22.11 ± 1.66 mg Pb^2+^/L, 27.20 ± 1.74 mg Pb^2+^/L, and 34.20 ± 1.80 mg Pb^2+^/L, respectively [33]. Singh and Manjeet (2015) argued that the differences were due to various factors such as age, sex, diet, material composition, and experimental conditions, as well as species [34]. In this study, the 96 h LC_50_ of the *P. stellatus* by waterborne Pb was relatively high at 226.882 mg Pb^2+^/L. This is due to differences in the physiological and experimental environmental factors of the fish, suggesting that *P. stellatus* has a relatively high tolerance limit to waterborne lead compared to other fish species. In future research, advanced studies should be conducted by comprehensively considering biological and water quality environmental factors, experimental conditions, etc., for accurate toxicity assessment after acute exposure to waterborne Pb.

Toxic substances can damage the gill epithelium, causing gas exchange disorders, or directly affect hematological characteristics by causing red blood cells (RBCs) circulating damage, premature death, and hemolysis through the circulatory system [35]. According to Habib et al. [36], hematological parameters in fish are sensitive to the toxic effects of metal exposure and reflect physiological changes in the fish body, so they can be used as an indicator to evaluate the toxic effects of waterborne Pb exposure on aquatic organisms. Vo et al. (2021) reported a significant decrease in hematocrit, hemoglobin, and RBC counts in red tilapia, *Oreochromis* sp., exposed to waterborne Pb [37]. In addition, an increase in MCV was observed as the RBC decreased, which was due to RBC swelling caused by waterborne Pb exposure. The increase in the MCH and MCHC was attributed to erythrocyte lysis and iron loss, which explained the decrease in hemoglobin concentration by waterborne Pb exposure. Saeed et al. (2024) also reported a decrease in hematocrit and hemoglobin in grass carp, *Ctenopharyngodon idella*, exposed to waterborne Pb, which was attributed to hemodilution due to impaired osmoregulatory control or gill damage [38]. Çiftçi et al. (2015) reported a significant increase in hematocrit and MCV in European eel, *Anguilla anguilla*, exposed to waterborne Pb for 7, 15, and 30 days, which was attributed to the stimulation of erythropoiesis by hemoconcentration or feedback mechanisms [39]. Ates et al. (2008) also argued that the decrease in hematocrit, hemoglobin, and RBC counts of rainbow trout, *Oncorhynchus mykiss*, exposed to waterborne Pb was due to the inhibition of erythrocyte production in the hematopoietic organ [40]. The MCV of *O. mykiss* significantly increased due to waterborne Pb exposure, while no changes in MCH and MCHC were observed. They argued that this was due to macrocytic anemia caused by waterborne Pb exposure.

On the other hand, Ergonul et al. (2012) reported that the RBC counts and hemoglobin of *C. carpio* significantly increased, while no significant changes in hematocrit were observed [41]. They argued that this was due to the increase in new erythrocytes to recover erythrocyte damage and oxygen carrying capacity reduction due to waterborne Pb exposure, early death of mature erythrocytes, and promotion of hemoglobin synthesis. Ciftci et al. (2008) reported that hematocrit of *A. anguilla* exposed for 15 and 30 days showed a significant increase, suggesting that this was due to the release of erythrocytes from erythropoietic tissues stimulated by waterborne Pb exposure [42]. In this study, the hemoglobin, hematocrit, and RBC counts of the *P. stellatus* significantly decreased after 96 h of acute exposure to waterborne Pb, which was thought to be due to anemia caused by waterborne Pb entering the circulatory system through the gills, damaging RBCs in the gills, inhibiting RBC production, and inducing hemolysis. In this study, the MCHC of the *P. stellatus*, analyzed based on the results for hemoglobin concentration, hematocrit value, and RBC count, showed a significant increase due to 160 mg Pb^2+^/L, but no significant changes were observed in MCV, which was due to hemoconcentration or a decrease in hemoglobin concentration caused by exposure to a high level of waterborne Pb of 160 mg Pb^2+^/L. As reported in [43], similarly, MCH decreased at 20 mg Pb^2+^/L, likely due to the production of immature RBCs with lower hemoglobin content, whereas at 160 mg Pb^2+^/L, severe Pb toxicity induced increased hemolysis due to the compensatory mechanism, leaving fewer but more hemoglobin-rich erythrocytes, thereby increasing MCH.

Of the inorganic plasma components, calcium performs physiological functions for the growth and maintenance of the skeletal system in fish, as well as for maintaining homeostasis [44]. Waterborne Pb can induce ion regulation dysfunction, gill Ca^2+^ influx, and homeostasis in fish, and it can be used as an indicator of toxic effects on the physiological and renal functions of fish due to waterborne Pb exposure [45,46]. Srivastav et al. (2013) reported a significant decrease in the plasma calcium levels of freshwater catfish, *Heteropneustes fossilis*, exposed to waterborne Pb for 96 h and 28 days, which was due to hypocalcemia caused by the decreased electrolyte influx through the gills or impaired renal function [47]. Ribeiro et al. (2014) reported a decrease in plasma calcium levels in streaked prochilods, *Prochilodus lineatus*, exposed to waterborne Pb, which may be involved in the inhibition of calcium ion transport during the process of inflow into the bloodstream by the metabolism of *P. lineatus* using the Na^+^/Ca^2+^ exchanger or ATP-dependent calcium pumps [48]. Atli and Canli (2007) suggest that the influx of waterborne Pb into fish occurs through a similar mechanism as the transport of Ca^2+^, resulting in increased claims of plasma Ca^2+^ being impaired [49]. Lagrana et al. (2011) also argued that competition between metal ions and calcium ions in Ca^2+^ transport could inhibit ion exchange [50]. In this study, waterborne Pb exposure significantly decreased plasma calcium in the *P. stellatus*, which was due to hypocalcemia caused by the decreased Ca^2+^ influx into the fish body due to competition between Pb^2+^ and Ca^2+^.

Plasma glucose not only regulates osmotic pressure in fish but also maintains hydromineral balance, and it is widely used as an indicator of fish stress [51]. Exposure to heavy metals causes stress in fish [52]. In response to stress, cortisol secretion in fish increases, which can promote glycogen breakdown and gluconeogenesis, leading to an increase in plasma glucose [53,54]. Kumar et al. (2017) reported that plasma glucose levels in *P. hypophthalmus* increased following exposure to waterborne Pb, which was due to the breakdown of glycogen in fish to generate energy to cope with stress caused by heavy metal exposure [55,56]. Vinodhini and Narayanan (2009) reported that plasma glucose levels in *C. carpio* exposed to metals, including waterborne Pb, increased, which was attributed to the stimulation of glycogen breakdown in the fish, thereby leading to hyperglycemia [57]. Additionally, Nirmalan and Nirmalan (2020) reported the increase in glucose levels may be attributed to the stimulation of glycogenolysis or the disruption of pancreatic beta cells, which are responsible for insulin production [58]. Meanwhile, Hajirezaee et al. (2021) claimed that waterborne lead exposure promoted glycogenolysis in grey mullet, *Mugil cephalus*, which depleted glycogen in the fish liver and decreased plasma glucose levels [59]. Kaya et al. (2015) also reported that plasma glucose levels in Mozambique tilapia, *Oreochromis mossambicus*, decreased from waterborne Pb exposure, which was attributed to the stimulation of carbohydrate metabolism by the Pb toxicity [60]. However, in this study, no significant changes in plasma glucose in the *P. stellatus* were observed as a result of waterborne Pb exposure.

Plasma cholesterol is a component of the outer layer of cell membranes and plasma lipoproteins and is a precursor of steroid hormones [61]. Metals can damage the liver parenchyma of fish, impairing their ability to metabolize cholesterol, which can lead to cholesterol excretion from the damaged liver, thereby increasing plasma cholesterol levels [62]. Giri et al. (2018) reported an increase in plasma cholesterol levels in *C. carpio* exposed to waterborne Pb, which they attributed to liver and kidney damage [63]. Oluah et al. (2014) reported an increase in plasma cholesterol in African sharptooth catfish, *Clarias gariepinus*, exposed to waterborne Pb, which was attributed to the disturbance of cholesterol metabolism in the liver [64]. On the other hand, Atli et al. (2015) reported that waterborne Pb exposure decreased plasma cholesterol levels in Nile tilapia, *Oreochromis niloticus*, which was attributed to inhibition of cholesterol synthesis due to cell membrane damage following waterborne Pb exposure [65]. Tunçsoy et al. (2015) reported that short-term waterborne Pb exposure increased plasma cholesterol levels in *C. gariepinus*, but the cholesterol levels decreased with increasing exposure period [66], which was due to tissue damage caused by waterborne lead toxicity, inhibition of cholesterol synthesis, or cholesterol use for the synthesis of steroid hormones. However, no significant changes in the plasma cholesterol of the *P. stellatus* were observed following exposure to waterborne Pb in this study.

Total plasma protein is a sensitive indicator of stress and hepatic damage in fish, responding to environmental stressors such as Pb exposure [67,68]. Total plasma protein in fish exposed to metals may decrease due to structural denaturation and weakening of proteins, inhibition of production, etc. [69]. Ayyat et al. (2020) reported that plasma total protein in *O. niloticus* decreased, which was attributed to the excretion of protein through urine due to renal dysfunction caused by the Pb toxicity [70]. Sahiti et al. (2018) also reported that waterborne Pb exposure decreased plasma total protein in *C. carpio*, which was attributed to protein degradation due to stress caused by the metal toxicity [71]. Ciftci et al. (2008) argued that the plasma total protein level may decrease due to increased synthesis of metal binding proteins in *A. anguilla* by waterborne Pb exposure [42]. On the other hand, Habib et al. (2024) reported an increase in plasma total protein in *C. carpio* exposed to waterborne Pb depending on exposure concentration and exposure period [36]. However, Nourian et al. (2019) reported that there were no significant changes in plasma total protein, cholesterol, and glucose in *C. carpio* exposed to Pb for 15 days [72]. Fırat et al. (2011) also reported that there were no significant changes in plasma total protein in *O. niloticus* exposed to Pb for 4 days, and significant changes occurred after 21 days of exposure [73]. In this study, no significant changes were observed in plasma total protein and plasma organic components of the *P. stellatus* from Pb exposure, which was thought to be the result of differences in experimental factors such as exposure time, exposure concentration, or experimental fish species [74,75].

AST and ALP are enzymes that play an important role in protein and amino acid degradation metabolism [65]. They are sensitive indicators for evaluating toxic effects on the liver, pancreas, muscles, and gills of fish, being released into the plasma following tissue damage and dysfunction due to environmental stress such as metal exposure [76]. Hwang et al. (2016) reported an increase in plasma AST and ALP levels in *P. stellatus* due to dietary Pb exposure, which was attributed to intracellular enzyme leakage or disruption of the enzyme transportation system by cell membrane deformation caused by Pb degeneration and necrosis of liver tissue [77]. Reddy et al. (2023) also claimed that waterborne Pb exposure induced liver tissue necrosis in *P. hypophthalmus*, which was attributed to enzyme leakage into the blood, leading to an increase in plasma AST and ALP levels [43]. Meanwhile, Çoğun et al. (2013) reported a decrease in plasma AST of *O. niloticus* following waterborne Pb exposure [78]. Mutlu (2016) also reported that plasma AST and ALP of *C. carpio* exposed to waterborne Pb for 30 and 90 days decreased, which could lead to muscle tissue degeneration [79]. In this study, plasma AST of the *P. stellatus* significantly decreased following waterborne Pb exposure, while no significant change in ALP was observed, which was thought to be due to the inhibition of plasma enzyme AST production by severe liver cell damage.

This study highlights important toxic effects of waterborne Pb exposure on *P. stellatus*, but it also has some limitations that need to be addressed in future work. The exposure duration of 96 h primarily examined acute toxicity, and longer exposure periods are necessary to evaluate long-term effects and accumulation of Pb. Additionally, the controlled laboratory conditions used in this study may not fully replicate natural environmental factors such as temperature fluctuations, pH, or salinity, which can alter the toxicity of Pb. Future research should aim to integrate these environmental variables and explore the combined impact of other pollutants commonly found in aquatic habitats. Investigating the responses of different species and utilizing advanced biomarkers could enhance our understanding of the underlying mechanisms of Pb toxicity. Expanding research in these areas will contribute to more accurate risk assessments and help in developing effective strategies to manage Pb contamination in aquatic environments.

## 5. Conclusions

In conclusion, high levels of waterborne Pb exposure caused significant mortality in *P. stellatus*, with the 96 h LC_50_ determined to be 227 mg Pb^2+^/L, indicating a relatively high tolerance compared to other fish species. Waterborne Pb toxicity significantly decreased hematological parameters (Ht, Hb, and RBC counts) in *P. stellatus*. Additionally, Pb exposure affected plasma calcium and AST levels. In contrast, no significant changes were observed in plasma glucose, cholesterol, and total protein levels. These findings suggest that the physiological characteristics of *P. stellatus* and experimental conditions influenced the outcomes. Further research considering biological and environmental factors is necessary for a more accurate assessment of waterborne Pb toxicity.

## Figures and Tables

**Table 1:** The chemical components of water and experimental condition used in the experiments.

Item	Value
Temperature (°C)	17.5 ± 0.3
pH	7.9 ± 0.1
Salinity (‰)	31.1 ± 0.1
Dissolved Oxygen (mg/L)	6.3 ± 0.3
Ammonia (µg/L)	17 ± 2.3
Nitrite (µg/L)	6.3 ± 0.8
Nitrate (µg/L)	0.12 ± 0.01
Phosphate (µg/L)	0.2 ± 0.02

**Table 2:** Lethal concentration (LC_50_) of starry flounder, *Platichthys stellatus*, exposed to waterborne lead for 96 h.

95% Confidence Limits	
Probability	Estimate (mg/L)
0.01	160.068
0.10	190.075
0.20	202.710
0.30	211.821
0.40	219.606
0.50	226.882
0.60	234.158
0.70	241.943
0.80	251.054
0.90	263.689
0.99	293.696

## Data Availability

Data are contained within the article.
